# Rapid Myoglobin Aggregation through Glucosamine-Induced α-Dicarbonyl Formation

**DOI:** 10.1371/journal.pone.0139022

**Published:** 2015-09-25

**Authors:** Yuliya Hrynets, Maurice Ndagijimana, Mirko Betti

**Affiliations:** Department of Agricultural, Food and Nutritional Science, University of Alberta, Edmonton, Alberta, Canada; Chi-Mei Medical Center, TAIWAN

## Abstract

The extent of glycation and conformational changes of horse myoglobin (Mb) upon glycation with *N*-acetyl-glucosamine (GlcNAc), glucose (Glc) and glucosamine (GlcN) were investigated. Among tested sugars, the rate of glycation with GlcN was the most rapid as shown by MALDI and ESI mass spectrometries. Protein oxidation, as evaluated by the amount of carbonyl groups present on Mb, was found to increase exponentially in Mb-Glc conjugates over time, whereas in Mb-GlcN mixtures the carbonyl groups decreased significantly after maximum at 3 days of the reaction. The reaction between GlcN and Mb resulted in a significantly higher amount of α-dicarbonyl compounds, mostly glucosone and 3-deoxyglucosone, ranging from and 27 to 332 mg/L and from 14 to 304 mg/L, respectively. Already at 0.5 days, tertiary structural changes of Mb-GlcN conjugate were observed by altered tryptophan fluorescence. A reduction of metmyoglobin to deoxy-and oxymyoglobin forms was observed on the first day of reaction, coinciding with the greatest amount of glucosone produced. In contrast to native α-helical myoglobin, 41% of the glycated protein sequence was transformed into a β-sheet conformation, as determined by circular dichroism spectropolarimetry. Transmission electron microscopy demonstrated that Mb glycation with GlcN causes the formation of amorphous or fibrous aggregates, started already at 3 reaction days. These aggregates bind to an amyloid-specific dye thioflavin T. With the aid of α-dicarbonyl compounds and advanced products of reaction, this study suggests that the Mb glycation with GlcN induces the unfolding of an initially globular protein structure into amyloid fibrils comprised of a β-sheet structure.

## Introduction

Non-enzymatic modification of proteins through the Maillard reaction has recently gained new attention. This process produces neoglycoproteins with enhanced functionality (i.e. solubility, emulsification capacity, etc.) and melanoproteins with improved antioxidant activity [1,2]. The Maillard reaction is a complex reaction between protein and reducing sugars and can be reduced to three major steps; the formation of a keto- or aldosamine (Amadori or Heyns compound, respectively), an intermediate reactive compound like α-dicarbonyl compounds (α-DC), and a final stage where yellow and brown condensation products are formed (heterocyclic compounds and melanoidins/melanoproteins, respectively). Fogliano and Moralez [3] reported that these melanoproteins can be divided into soluble and insoluble protein aggregates. Insoluble protein aggregates are resistant to protein digestion and would possibly represent a dietary fiber. However, to date, there are few studies reporting the nature of the Maillard protein aggregates in food systems. On the other hand, several *in vitro* studies have simulated physiological conditions to demonstrate the effect of high level of sugars or α-DC like glyoxal and methylglyoxal, on protein modification [4,5,6]. Amyloid fibrils are insoluble protein aggregates deposited extracellularly in tissues that have a pathogenic effect leading to amyloid-like diseases including Alzheimer's disease, and the spongiform encephalopathies [7]. Furthermore, extensive islet amyloid formation is induced by development of Type II diabetes mellitus and contributes to its progression [8]. Based on these considerations, it is important to distinguish the effect of the endogenous and exogenous Maillard reactions. The endogenous reaction may form modified protein structures that affect cell metabolism, whereas the exogenous reaction occurs during food processing and confers positive qualities regarding flavour, colour, antimicrobial activity [9] and also antioxidant capacity [10,11]. Beside these positive effects, there is a controversy on the harmful consequences that some of the Maillard reaction products, advanced glycation end-products (AGEs) in particular, exert on human health. Well known examples of these compounds are acrylamide and 5-hydroxymethylfurfural, also referred as neo-formed contaminants [12].

Glucosamine (GlcN) is an amino monosaccharide used by consumers as a dietary supplement to both reduce osteoarthritis pain and improve joint function [13]. GlcN supplementation may stimulate synovial production of hyaluronic acid (HA), a type of glycosaminoglycan responsible for the lubricating and shock-absorbing properties of synovial fluid in cartilage [14]. In a previous study [15] 1 g of GlcN produces up to 6 mg of reactive α-DC, mainly glucosone and 3-deoxyglucosone during incubation at 37°C from 0 to 12 days. This because GlcN is a Heyns compound that, like the Amadori compound (fructosamine), can degrade rapidly under certain conditions. By contrast, the amide form of GlcN, *N*-acetyl-GlcN (GlcNAc), does not produce a significant amount of α-DC due to the stabilizing effect of the acetyl group on the nucleophilicity of the -NH_2_ group. The possibility of producing a significant amount of the Heyns compound with a “simple” deacetylation process allows for an opportunity to design a model system to study how a first product of the Maillard reaction interacts with protein.

Equine myoglobin (Mb), a monomeric oxygen-binding protein found within muscle cells, was chosen since it is a readily available protein frequently used for *in vitro* studies and behaves similarly to Mb from meat-producing species [16]. This study serves to understand the effect of the degradation products of GlcN on Mb structure at 37°C incubation. The major changes in Mb structure were monitored by mass spectrometric (MALDI and ESI ionization), spectroscopic (circular dichroism and fluorescence) and microscopic techniques (TEM). GlcNAc and Glc were also incubated in the same conditions and used as comparison as reported in Hrynets et al. [15].

## Materials and Methods

### Materials

Myoglobin from equine skeletal muscle (95–100% purity, lyophilized powder), *N*-acetyl-D-glucosamine (≥99% purity), D-glucose (≥99.5% purity), D-glucosamine hydrochloride (≥99% purity), potassium phosphate monobasic and dibasic, sodium azide, HPLC-grade solvents (acetonitrile, methanol, formic acid, trifluoroacetic acid), glucosone (2-keto-D-glucose; ≥98.0% purity; M_W_ 178.14 Da), glyoxal (ethanedial; 40% in H_2_O; M_W_ 58.04 Da), methylglyoxal (2-oxopropanal; 40% in H_2_O; M_W_ 72.06 Da), diacetyl (butane-2,3-dione; ≥95.0% purity; M_W_ 86.09 Da), 1,2-diaminobenzene, DTPA and Thioflavin T were purchased from Sigma-Aldrich (St. Louis, MO). 3-deoxyglucosone (3-Deoxy-D-erythro-hexosulose; ≥95% purity; M_W_ 162.14 Da) was obtained from Cayman Chemical (Ann Arbor, MI). SPE tC-18 Sep-Pak Vac 6 cc columns were obtained from Waters (Milford, MA). Filtration membranes (0.22 μm) were from Millipore (Billerica, MA). 3,5 dimethoxy-4-hydroxycinnamic acid (sinapinic acid), ProMix3 (mass spectrometry protein standards) were obtained from LaserBiolabs. Standard ion calibration solution (Pierce LTQ) for electrospray ionization in positive mode was purchased from Thermo Scientific (Rockford, IL, USA). Milli-Q purified distilled water for buffers and reagents preparations were purchased from Waters Millipore (Milford, MA, USA). All the reagents and chemicals used in the study were of analytical grade.

### Experimental design

The non-enzymatic modifications of Mb in presence of GlcNAc, Glc and GlcN were evaluated over time at 0, 0.5, 1, 2, 3, 6 and 12 days in phosphate buffer at 37°C. In the first part of the study, mass spectrometric profiles were assessed over time to understand the major changes occurring during the reaction as dependent on the type of monosaccharide. In this regard, a total of 42 tubes (2 tubes × monosaccharide × incubation time) were randomly placed within the incubator and the reaction was monitored over time. At each time, 2 tubes per treatment were pooled and subjected to MS analysis. Next, α-dicarbonyl content was evaluated, where 3 tubes per treatment were incubated, resulting in 63 samples in total. Then 9 tubes per treatment were incubated, from which 3 tubes were used for Trp fluorescence analyses, 3 tubes to assess protein oxidation level and 3 tubes for circular dichroism. Finally, Thioflavin T fluorescence analyses were performed for all treatments and selected incubation times were used for microscopy.

### Preparation of glycoforms of myoglobin

For the preparation of myoglobin-sugar conjugates, 5 mg/mL of horse skeletal muscle myoglobin was incubated at 37°C (Thermoshaker Innova 44, New Brunswick Scientific, USA) in the presence of GlcNAc, Glc or GlcN (1:3 ratio, w/w) in 50 mM potassium phosphate buffer (pH 7.4) containing 0.02% NaN_3_ as bacteriostat. Final pH values were adjusted to 7.4 when necessary. The controls were only protein and only sugars solutions at zero time and incubated. The samples aliquots were transferred into plastic screw-cap aliquot tubes and randomly arranged within an incubator (New Brunswick Scientific, Edison, NJ). After collection at 0.5, 1, 2, 3, 6 and 12 d, the samples were ultrafiltrated with Amicon Ultra 3K membrane (molecular weight cut-off of 3.000 NMWL; Millipore Corporation, Bedford, MA, USA) to eliminate unreacted sugar. Incubations did not fluctuate by more than 0.1 pH units up to 12 days (Mb-Glc -NH_3_) or 0.4 units (for Mb-GlcN). The ultrafiltration step was avoided for the samples subjected to the analyses of free α-dicarbonyl compounds and transmission electron microscopy.

### Matrix assisted laser desorption/ionisation time-of-flight mass spectrometry (MALDI-TOF-MS)

MALDI-TOF-MS analyses were performed on a Bruker Ultraflextreme MALDI-TOF/TOF mass spectrometer (Bruker, Bremen, Germany) using positive ionization linear ion mode between 5 and 20 kDa and sinapinic acid as a matrix. The sample preparation protocol for MALDI-TOF/TOF MS is described as follows: a solution of the protein sample (1 μL) was spotted on a ground steel MALDI plate and acidified by spotting 1 μL of 0.1% trifluoroacetic acid (TFA) in water on top and air dried. A 1 μL aliquot of a stock solution of sinapinic acid (10 mg/mL) in 50% acetonitrile (ACN)/ 50% H_2_O was spotted on top and air dried. Prior to each experiment the apparatus was calibrated using a reference protein solution (Sigma-Aldrich, St. Louis, MO). Data analysis was performed using the Bruker flex Analysis software package version 3.3.

### Liquid chromatography—electrospray ionization mass spectrometry (LC-ESI-MS)

For protein molecular weight determination reverse phase high performance liquid chromatography followed by detection using ultraviolet absorption and mass spectrometry (RP-HPLC-UV-MS) was performed using an Agilent 1200 SL HPLC System with a Poroshell 300SB-C8, 5 micron particle size, 75 x 0.5 mm column (Agilent Technologies, USA), with Opti-pak trap cartridge kit, 5 μL BED, C8, thermostated at 60°C. A buffer gradient system composed 0.1% formic acid in water as mobile phase A and 0.1% formic acid in ACN as a mobile phase B. An aliquot of 5 μL of sample was loaded onto the column at a flow rate of 0.15 mL/min and an initial buffer composition of 95% mobile phase A and 5% mobile phase B. After injection, the column was washed using the initial loading conditions for 3 minutes to effectively remove salts. Elution of the proteins was done by using a linear gradient from 5% to 60% mobile phase B over a period of 15 minutes, 60% to 80% mobile phase B over a period of 3 min, 80% to 98% mobile phase B over a period of 2 min. UV absorbance was monitored at 210, 214, 254 and 280 nm. Mass spectra were acquired in positive mode of ionization using an Agilent 6220 Accurate-Mass TOF HPLC-MS system (Santa Clara, CA, USA) equipped with a dual sprayer electrospray ionization source with the second sprayer providing a reference mass solution. Mass correction was performed for every individual spectrum using peaks at m/z 121.0509 and 922.0098 from the reference solution. Mass spectrometric conditions were drying gas 10 L/min at 325°C, nebulizer 20 psi, mass range 100–3000 Da, acquisition rate of ~1.03 spectra/sec, fragmentor 200 V, skimmer 65 V, capillary 3200 V, instrument state 4 GHz High Resolution. Data analysis was performed using the Agilent MassHunter Qualitative Analysis software package version B.03.01 SP3.

### Assay of carbonyl formation

To understand glycation-induced carbonyl stress, carbonyl formation in Mb and glycated Mb forms was detected by reactivity with 2,4-dinitrophenylhydrazine (DNPH) to form protein hydrazones according to Chan et al. [17]. Control and conjugated solutions were diluted to a protein concentration ranging from 0.7 to 1.0 mg/mL and precipitated with 10% TCA (w/v). After centrifugation (2000*g*, 10 min, 4°C) the blank was treated with 4 mL of 2 M HCl and the test samples (pellet) with 4 mL of 0.2% DNPH (w/v) in 2 M HCl. The tubes were incubated for 1 h at 25°C in the dark with agitation every 10 min. After precipitation with 10% TCA, the solutions were further centrifuged to collect the protein precipitates. Next, the pellet was washed twice with 1 mL of ethanol/ethyl acetate (1:1, v/v), precipitated with 10% TCA and then centrifuged. The final precipitate was dissolved in 2 mL 6 M guanidine hydrochloride in 20 mM phosphate buffer (pH 6.5). The carbonyl group content was determined spectrophotometrically (V-530, Jasco Corporation, Tokyo, Japan) at a wavelength of 365 nm for the DNPH-treated samples against an HCl control. Protein concentration was calculated from the absorbance at 280 nm in the HCl control using a standard bovine serum albumin (BSA) in guanidine. The carbonylation level was calculated using absorption coefficient of 22,000 M^−1^ cm^−1^ for protein hydrazones and expressed as nmol of DNPH/mg of protein.

After subtraction of zero time values, the mean values were then plotted and fitted (except Mb-GlcN treatments) with the non-linear fitting using GraphPad Prism software (version 4.0, San Diego, CA, USA).

### Extraction of α-dicarbonyl compounds

The extraction method was based on a three-step procedure described by Papetti et al. [18]. Briefly, collected aliquots of Mb-GlcNAc, Mb-Glc and Mb-GlcN (6.0 mL) were passed through a pre-conditioned SPE tC-18 Sep-Pak cartridge (Waters, Milford, MA, USA) at a flow rate close to 2 mL/min as a clean-up step before derivatization. Collected polar fraction was spiked with 0.006 g of 1,2-diaminobenzene (*O*-phenylenediamine (*o*-OPD)), the pH was adjusted to 3.00 ± 0.02 with HCl (4 N), and the fraction was derivatized at 37°C for 1 h in the presence of 11 mM diethylene triamine pentaacetic acid (DTPA) [19]. The quinoxaline derivatives were eluted from another SPE cartridge with 4 mL of a MeOH/H_2_O mixture (90/10 v/v). The first 1 mL was discarded, whereas the next 2 mL were used for analysis.

### Separation of the quinoxalines derivatives of α-dicarbonyl compounds by UHPLC-UV

α-DC analysis was conducted using an Ultrahigh Performance Liquid Chromatography apparatus (Shimadzu, Columbia, MD) consisting of two pumps LC-30AD, an SPD-M20A photo diode array detector (PDA), DGU–20A5 degasser, SIL–30AC autosampler (operated at 4°C) and CTO-20 AC column oven. The UHPLC system was controlled by a personal computer equipped with LabSolutions software operating in a Windows XP. The separation was achieved following the method described by Papetti et al. [18] using Ascentis Express ES-C18 column (150 × 4.6 mm, 2.7 μm particles; Sigma-Aldrich, St. Louis, MO) with a UHPLC pre-column filter (UltraShield Analytical Scientific Instruments, Richmond, CA) operating at 25.0 ± 0.5°C. The analytes were eluted with a flow rate of 0.3 mL/min using 0.1% formic acid in water (A) and methanol (B) as eluents. The 120 min gradient is described as follows: 0–5 min (90–85% A), 5–13 min (85–80% A), 13–40 min (80% A), 40–65 min (80–70% A), 65–90 min (70–50% A), 90–100 min (50–0% A), 100–105 min (0% A), and 105–110 min (0–90% A); the column was then re-equilibrated with the initial mobile phase for 10 min. The injection volume was 5 μL. The PDA detector was set at 314 and 335 nm to record the peaks, and UV spectra recorded from 215 to 420 nm. Before injection sample solutions were filtered with PVDF syringe filter (13 mm, 0.22 μm; Millipore Millex, Billerica, MA, USA).

### Identification and quantitation of quinoxaline derivatives

The identification of major α-DC was based on comparison of their retention times of the reference compounds (quinoxalines derivatives of G, 3-DG, GO, MGO and DA) and spectral characteristics. For unequivocal identification of the major α-DC, eight UHPLC peaks were manually collected and subjected to mass spectrometry analyses. The accurate mass and MS/MS fragmentation patterns were compared to authentic standards and to the reference data. Mass spectrometry analyses were performed in duplicates.

For quantitation external calibration was used. Each quinoxaline derivative was diluted to the final concentrations ranging from 60–2000 (G), 1.5–2000 (3-DG), 4.5–200 (GO), 1.0–25 (MGO), and 1.25–12 μM for DA. Each concentration was analyzed in triplicate. The peak area was plotted against concentration and the regression equations were calculated. The correlation coefficients for all calibration curves were R^2^ ≥0.99. The limit of detection (LOD) was calculated as 2.08 (G), 0.26 (3-DG), 0.13 (GO), 0.09 (MGO) and 0.20 μM (DA). The limit of quantification (LOQ) was determined as 6.30 (G), 0.78 (3-DG), 0.40 (GO), 0.28 (MGO), and 0.61 μM (DA) by assuming a signal-to-noise ratio (S/N) 3:1 for LOD and S/N 10:1 for LOQ.

### Direct infusion orbitrap mass spectrometry analyses (DIMS)

Collected α-DC fractions from UHPLC were subjected to DIMS. All Orbitrap measurements were carried out by using the Ion Max electron spray ionization (ESI) source (Thermo Fisher Scientific) mounted on a LTQ Orbitrap XL (Thermo Scientific, San Jose, CA, USA). The orbitrap mass analyzer was calibrated with standard ion calibration solutions (Pierce LTQ, Thermo Scientific). Following parameters were applied: sheath gas flow 15 arbitrary units, auxiliary gas flow 5 arbitrary units, and capillary temperature of 275°C. Samples (3 μL) were injected manually performing direct infusion using 100 μL syringe (Hamilton, Reno, NV, USA) and the on board syringe pump. DIMS analyses were performed at a mass resolution of 60.000 at m/z 400 and spectra were acquired over the range of m/z 50–500. The automatic gain control (AGC) target was set to 1e6 and the maximum injection time to 250 ms. For MS/MS analyses, ions of interest were isolated with an isolation width of 1–2 Da and collision induced dissociation was conducted at varying voltages which were optimized for each sample. An activation time of 30 ms was used with a tube lens voltage of 110 V and a capillary temperature of 275°C. Data acquisition and processing were performed using Xcalibur software (Thermo Scientific).

### Determination of myoglobin forms

Control and glycoconjugated samples were diluted to 1 mg/mL with 50 mM potassium phosphate buffer (pH 7.4) and the absorption was recorded at 557, 582, 503 and 525 nm using a Shimadzu UV-2101 spectrophotometer (Kyoto, Japan) with cuvettes of 1 cm path length. The relative proportions of the myoglobin redox forms: deoxymyoglobin (DeoMb), oxymyoglobin (OxyMb) and metmyoglobin (MetMb) were calculated according to the modified Krzywicki’s equation described by Tang et al. [16] as follows:

$$\left[\text{DeoMb}\right]\;=\text{ - }0.543{\text{R}}_{1}+\;1.594{\text{R}}_{2}+\;0.552{\text{R}}_{3}–\;1.329$$

$$\left[\text{OxyMb}\right]\;=\;0.722{\text{R}}_{1}–\;1.432{\text{R}}_{2}–\;1.659{\text{R}}_{3}+\;2.599$$

$$\left[\text{MetMb}\right]\;=\text{ - }0.159{\text{R}}_{1}–\;0.085{\text{R}}_{2}+\;1.262{\text{R}}_{3}–\;0.520$$

where R_1_ = A_582_/A_525_, R2 = A_557_/A_525_ and R3 = A_503_/A_525_.

### Soret absorbance spectroscopy

Soret absorption of the heme group was monitored on a Spectramax M5 (Molecular Devices, Sunnyvale, CA) spectrofluorometer by using 1 cm path length cell. The collected samples were 20-fold diluted and the readings taken in the range of 380–430 nm.

### Circular dichroism (CD) spectra acquisition and analyses

Far-UV CD spectra of 0.2 mg/mL of unmodified and modified Mb in a 50 mM phosphate buffer (pH 7.4) were recorded at 20°C in the spectral range from 190 to 250 nm with spectral resolution of 2 nm on a OLIS DSM 17 UV-Vis-NIR CD spectrophotometer (Bogart, Georgia, USA). Spectra were recorded as averages of five scans. Quartz cuvettes with an optical path of 1 mm were used. The solvent reference (a protein-free buffer) spectrum was subtracted from each sample spectrum automatically after which the data were smoothed and converted to molar ellipticity (θ) units (deg·cm^2^/dmol^-1^), as described by Yang et al. [20]. The amount of secondary structure associated with samples was estimated using the CONTIN/LL program in CDPro software.

### Intrinsic fluorescence measurements

Recordings of fluorescence emission spectra were obtained immediately after incubation of control and treated solutions, diluted 20-fold, at specific time point. Spectra were recorded using quartzglass cuvettes (QS-1.000 Suprasil, HellmaGmbH & Co, Germany) (light path of 1 cm) at λ_excitation_ of 280 nm and λ_emission_ of 290–400 nm (Spectramax M5). Phosphate buffer (50 mM, pH 7.4) was used for samples dilution and baseline subtraction.

### Thioflavin T (ThT) fluorescence spectroscopy

ThT stock solution was prepared by dispersing 8 mg of ThT into 10 mL of 50 mM phosphate buffer (pH 7.4) containing 150 mM NaCl and passed through a 0.22 μm Millipore filter. This stock solution was stored in the dark at 4°C. The stock solution was diluted by 50-fold in the same buffer on the day of analysis to produce the working solution. To prepare samples for spectrofluorometry, 50 μL of samples aliquots were mixed with 5 mL of ThT working solution and allowed to stand for 1 min. The fluorescence spectra of the mixtures were acquired (Spectramax M5) using the excitation wavelength of 460 nm and the emission wavelength at a range of 480–550 nm [21]. The fluorescence spectrum of the ThT working solution and GlcN control were subtracted from the fluorescence spectra of the samples.

Followed subtraction of zero time values, data curves were plotted expressing the mean of triplicates with standard deviation. Curves were fitted with non-linear regression fit using GraphPad Prism software.

### Transmission electron microscopy (TEM)

Myoglobin fibrils formation in the presence of different sugars was monitored by TEM. Control and conjugated aliquots of 50 μL were sampled from a protein solution of 5 mg/mL, diluted 100-fold with phosphate buffer pH 7.4 and deposited on 400-mesh formvar/carbon grid (Ted Pella, Redding, CA) and allowed to absorb for 3 min. The excess liquid was blotted gently on Kimwipes (Kimberly-Clark). A drop of stain, 1% aqueous uranyl acetate (Ted Pella, Redding, CA) made up fresh was placed on the grid for 1 min. After drying on the bench, the grids were visualized by a Philips EM 410 at 80 kv.

### Statistical analyses

Data were tested for significance by analysis of variance (ANOVA) using the PROC MIXED procedure of SAS (v. 9.3, SAS Institute Inc., Cary, NC). Comparison among means was evaluated by performing Tukey’s honest significant difference (*p* < 0.05). Data for carbonyl groups content and ThT fluorescence were plotted as a function of incubation time to generate curves. Data were fitted with the non-linear fitting using GraphPad Prism software.

## Results

### Mass spectrometric glycoform profiling of myoglobin

#### MALDI-TOF/TOF-MS

The power of mass spectrometric techniques for the identification of glycated proteins has been widely accepted. MALDI and ESI mass spectrometries, in particular, are validated by numerous studies on protein glycation. The combinatorial approach with both techniques allows using the advantages typical for both techniques obtaining complimentary information and a better understanding of the reaction evolution. MALDI-TOF/TOF-MS was applied initially to investigate Mb’s *in vitro* glycation, performed by incubation at 37°C (pH 7.4) with GlcNAc, Glc and GlcN from 0 to 12 days. Modifications were monitored through the increase in the molecular weight as a result of covalent sugar or glycation product addition.

**Fig 1:**
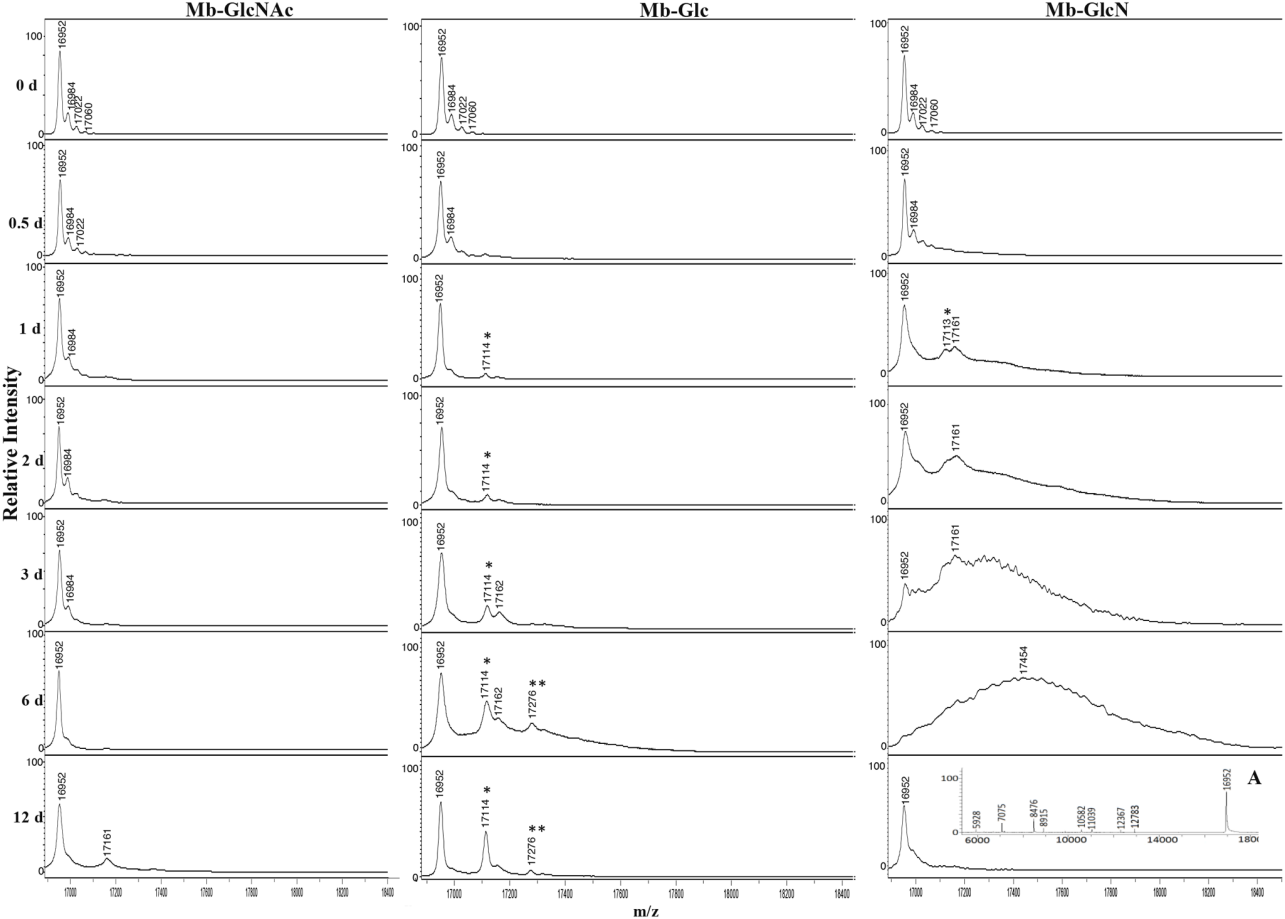
MALDI-TOF/TOF mass spectra of Mb incubated at 37°C for various times in the presence of GlcNAc, Glc and GlcN. Glycation adducts are marked with a star, with the number of stars corresponding to the number of adducts. Inset spectrum (A) shows the spectrum of GlcN incubated for 12 days in the region of m/z 6000–18000.

The MALDI spectrum from the different treatments is shown in Fig 1. The peak at m/z 16952, corresponding to a protonated apomyoglobin (apoMb) was found in all the spectra profiles. Heme-globin interactions are disrupted during desorption/ionization process resulting in extensive loss of the heme [22]. Other peaks presented in all the profiles were at m/z 16984, which corresponds to an increment of 32 Da possibly due to double oxidative adducts formation [23] and the peaks at m/z 17022 and 17060 are likely due to potassium adducts.

There were no major changes in the spectra Mb glycated with GlcNAc over time suggesting no glycation occurrence within the reaction period studied. The evaluation of blocked -NH_2_ groups (S1 Fig) supports this assumption, since only 3.9% of -NH_2_ was found to be modified at 12 reaction days, whereas for the Mb-Glc and Mb-GlcN treatments the percentage reached 32 and 49, respectively.

For Mb-Glc conjugates after 1 day of reaction a new peak at m/z 17114 arose, and corresponded to molecules produced by the condensation of one Glc molecule (+162 Da) [24]. With a prolonged reaction time the intensity of monoglycated peak increased and a peak at m/z 17276 was also observable starting from 6 reaction days, likely corresponding to a diglycated form with a mass shift of 324 Da [25].

In Mb-GlcN mixture after one reaction day GlcN monoadducts were identified at m/z 17113 (Δm = 161 Da). As the reaction progressed up to three days only broad and poorly resolved peaks were found, most likely due to both a large heterogeneity of the glycoforms [24] and modifications caused by the high production of α-DC generated from the non-enzymatic degradation of GlcN [15]. Furthermore, it is expected that in presence of proteins α-DC production would further increase (see α-DC section further). These extensive changes in MS profile were corroborated with the significant accumulation of AGEs (S2 Fig). By day 6 of reaction, only a large bell-shaped distribution of signal was observed and the disappearance of the signal from unmodified Mb, likely due to the increased formation of AGEs [26]. For instance, Zhang et al. [27] demonstrated accumulation of high molecular weight AGEs during glycation of fibrinogen and human serum albumin by GlcN at 37°C for 30 days.

Remarkably, slight protein precipitation was also observed by the naked eye after 3 days of glycation and became more significant after 6 and 12 days. Bokiej et al. [28] also reported un-interpretable MALDI mass spectra during the incubation of Mb with highly-reactive D-ribose-5-phosphate (R5P) for longer than 18 h.

Due to extensive precipitation of Mb-GlcN conjugates at 12 days, the MS analysis was mainly performed on the supernatant fraction, due to the inability to process the precipitated material. A significant protein fragmentation was observed, as evident from the appearance of smaller molecular weight fragments in the mass range m/z 6000–15000, apart from double charged ion peak of Mb at m/z 8476 (Fig 1A inset).

#### Electrospray ionization mass spectrometry (ESI-MS)

**Fig 2:**
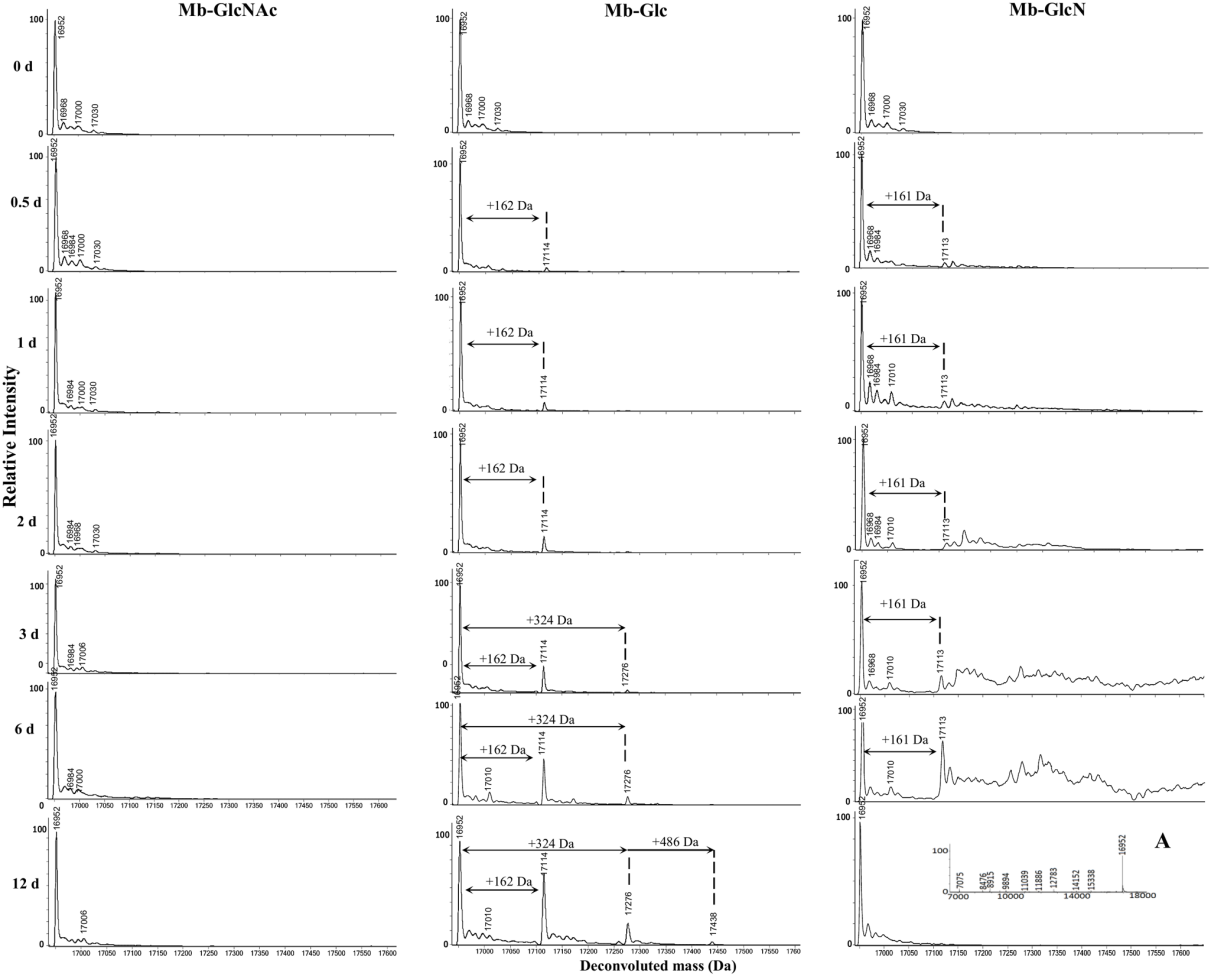
A deconvoluted ESI-MS spectra of Mb incubated at 37°C for various times in the presence of GlcNAc, Glc and GlcN. The experimental conditions were the same as those used to obtain the spectra in Fig 1. Inset spectrum (A) shows the spectrum of GlcN incubated for 12 days in the region of 7000–18000 Da.

The MALDI-TOF/TOF-MS results indicated extensive heterogeneity but no discrete masses could be resolved efficiently. To improve the analysis of modified Mb, the focus was shifted from MALDI-TOF/TOF-MS to LC/ESI-MS. A dramatic improvement of spectral resolution was obtained particularly for longer incubation periods. A typical ESI deconvoluted mass spectrum over time with different sugars is shown in Fig 2.

Similar to a MALDI-TOF/TOF-MS spectrum the major peak at 16952 Da was associated with apoMb. Raw data spectra for Mb-GlcNAc (0 and 12 days), Mb-Glc (0 and 12 days) and Mb-GlcN (0 and 6 days) are shown in S3, S4 and S5 Figs, respectively. Under ESI-MS analysis heme loss occurs, resulting in apoMb with a molecular weight of 16950±2 Da [29]. No GlcNAc addition was observed at the native protein mass + 203 Da (equivalent to one GlcNAc molecule) at any time in this study, confirming the findings from MALDI-TOF/TOF-MS. For Mb-Glc, a mass shift 162 Da upwards was observed incremental to the native apoMb already at 0.5 day and was assigned to a Glc (180 Da) adduct through elimination of water (−18 Da). The relative abundance of this peak at 17114 Da increased with incubation time, suggesting an increased concentration of sugar adducts over time. At 6 days of glycation the Mb’s molecular weight increased from 16952 Da to 17276 Da, corresponding to the condensation of two Glc units on Mb. The condensation of three Glc units was apparent by the peak at 17438 Da after 12 days of glycation. Both the increased abundance of glycated species and the presence of triglycated molecules clearly demonstrate the higher reactivity of glucose compared to GlcNAc.

The deacetylation of the GlcN -NH_2_ moiety makes it much more reactive towards Mb-GlcN conjugates, which showed progressively higher modifications over time. In the previous study [15] glucosone (G) and 3-deoxyglucosone (3-DG) were the most abundant α-dicarbonyl compounds produced during GlcN incubation. Glucosone (Mw = 178 Da) or 3-DG (Mw = 162 Da) attachment to amino acid residues in Mb could possibly result in the mass shift upward 160 Da or 144 Da, respectively. For instance, the resulting mass difference between GlcN (+161 Da) or G (+160 Da) attachment to Mb is only 1 Da, which is not enough for accurate peak assignment in an intact protein. The complexity of Mb-GlcN reaction mixture leads to a large varieties of glycation combinations, so the discussion here of peak origins is not definitive. Moreover the complexity of the raw data (S5 Fig) also complicates exact peaks identification. Starting from ½ days until 6 days the peak at 17113 Da was likely due to monoglycation (Δm = 161 Da). Many other peaks present in this spectra were attributed to the modification of Mb by various adducts, exact nature of which is unknown. The appearance of multiple peaks in the spectrum during prolonged incubation time refers to more numerous modifications, including the occurrence of oxidation/dehydration and possibly condensation [15] reactions in the presence of GlcN. As mentioned earlier, mostly the supernatant was analyzed at 12 days of reaction due to substantial protein precipitation. The ESI-MS results corroborate with those of MALDI-TOF/TOF-MS, showing strong protein degradation, generating fragments with a molecular weight ranging from 7000 to 16000 Da. Protein fragmentation is most likely due to the production of reactive oxygen species (ROS) generated in the Mb-GlcN model system [30]. In this regard, at least three major sources can contribute to ROS production. The first is direct autoxidation of GlcN [15] and the second is the Schiff base/Amadori compounds produced from both glycation of Mb with GlcN and α-DC condensation with Mb and GlcN, respectively [31]. Lastly, ROS production may be further facilitated by the presence of Mb, a biological Fenton reagent [32]. Human serum albumin scission to fragments of discrete sizes was also demonstrated over prolonged glycation in the presence of copper ions, proposed to cause fragmentation via free radical mechanism [33,34].

Although MALDI-TOF/TOF-MS results showed the same trend of the extent of glycation with different sugars, the superior peak resolution of ESI [35] was obtained. Thus in this regard, in our study conditions, ESI-MS was better than MALDI-TOF-MS, especially for Mb-GlcN conjugates with higher glyco-heterogeneity.

### Glycation induces myoglobin oxidative modifications through carbonyl formation

**Fig 3:**
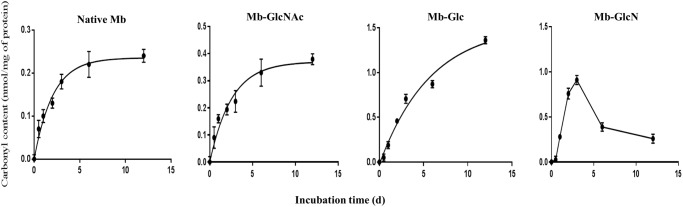
Protein oxidation (carbonyl content) in Mb and Mb conjugated with GlcNAc, Glc and GlcN from 0 to 12 days. The results are mean ± standard deviation of three independent experiments. Data were fitted (except Mb-GlcN) with the non-linear fitting by GraphPad Prism software using following exponential equation: *y* = A(1-e*^-kt^*), where *y* is the product concentration, A is the initial value at *t*_0_, *k* is the reaction rate, and *t* is time.

The Maillard reaction induces protein oxidation [36]. For instance, protein carbonylation is a type of protein oxidation that can be promoted by ROS, including hydrogen peroxide and free radicals, and usually refers to a process that forms reactive ketones or aldehydes [37]. Lysine, arginine, proline, and threonine residues, among other amino acids are normally subjected to direct ROS attack forming carbonyl groups [38]. As previously discussed in mass spectrometry section, it is expected that the reaction cocktail containing GlcN and Mb possesses all the triggers to produce an elevated concentration of ROS species. Fig 3 reports Mb carbonylation over time as a function of the different treatments. In the control (Mb incubated without reducing sugars) and GlcNAc treatments, carbonyl production increased slightly, reaching a plateau at concentrations of 0.23 and 0.37 nmol/mg protein, respectively. The same exponential fitting was applicable for Mb-Glc treatments withthe mean values of around 3.2 times higher as compared to Mb and Mb-GlcNAc, reaching a plateau at 1.56 nmol carbonyls/mg protein as estimated by the exponential fitting model. In addition, native Mb and Mb-GlcNAc mixtures showed a lower velocity of carbonyls development, as reflected by the plateau reached by 10–11 days of incubation, whereas in Mb-Glc conjugates the plateau level was reached at 12 days of glycation.

In Mb-GlcN treatment, carbonyls increased sharply up to 3 days incubation (1.01 nmol/mg protein), with a subsequent dramatic decrease (0.36 nmol/mg protein) at 6 days. This pattern was most likely due to the nature of GlcN which possess an -NH_2_ that can condense (and thus “quench”) with the carbonyl compounds on Mb. This also induces more glycation [37] with subsequent detrimental effects on Mb structure, thus leading to rapid protein aggregation (see section on conformational changes). Therefore, the higher reactivity of GlcN towards Mb compared to Glc and GlcNAc depends not only on the amount of α-DC produced at the beginning of the reaction (section α-DC formation), but also on the additional effect of the free -NH_2_ group of GlcN reacting with the carbonyls formed during Mb oxidation [39].

### Identification and quantitation of the major α-dicarbonyl compounds in Mb-GlcN

**Fig 4:**
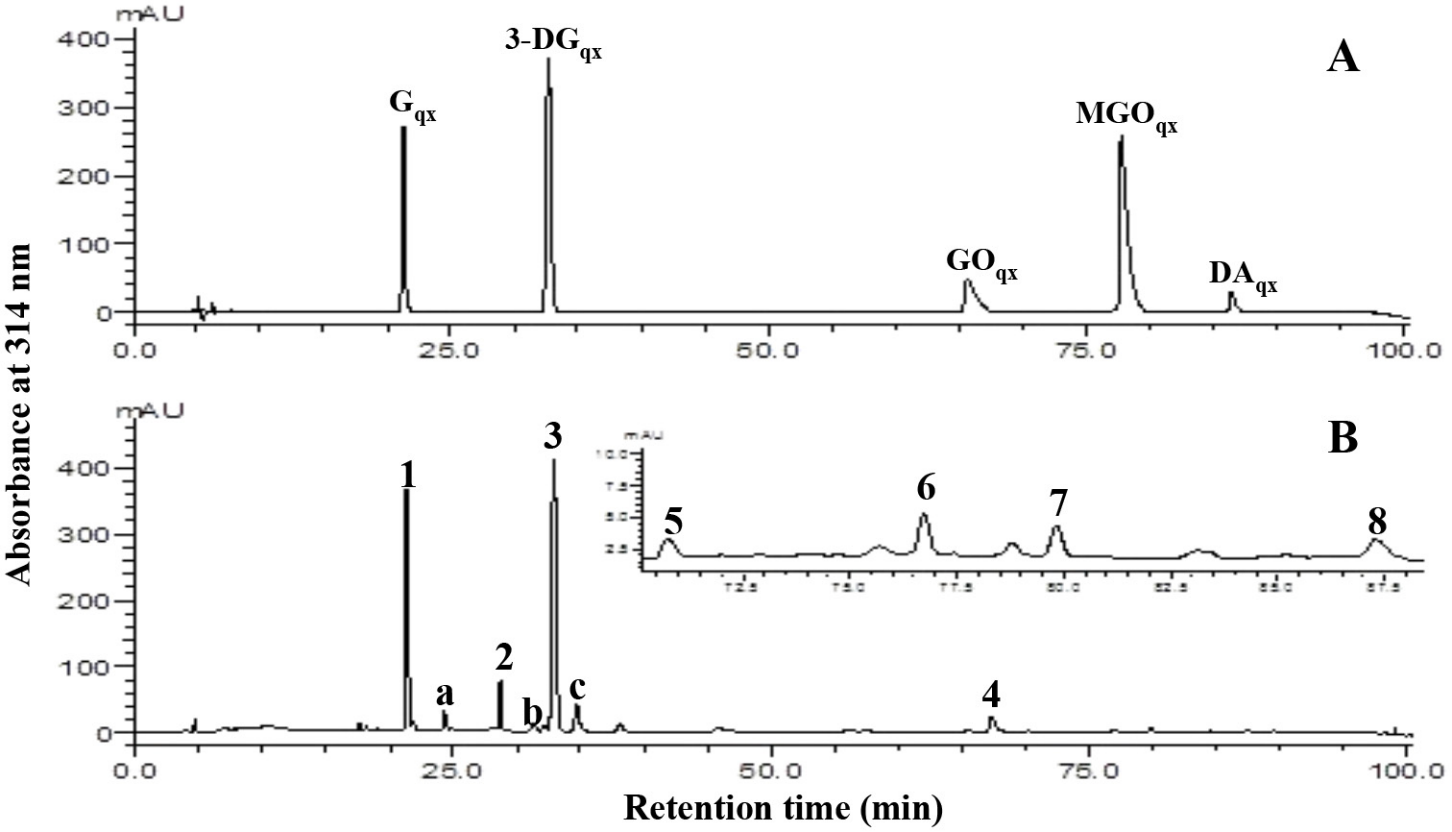
UHPLC analyses of quinoxaline derivatives of α-dicarbonyl compounds produced from Mb-GlcN conjugates over time. (A) Chromatograms of (I) a reference quinoxaline mixture of glucosone (G), 3-deoxyglucosone (3-DG), glyoxal (GO), methylglyoxal (MGO) and diacetyl (DA). (II) Representative chromatogram of Mb-GlcN conjugate incubated for 1 d, derivatized with *o*-OPD and acquired by UHPLC with UV detection at 314 nm. Numbers indicate the peaks of the quinoxalines of (1) G, (2) unidentified, (3) 3-DG, (4) GO, (5) HPA, (6) 3,4- DGE, (7) MGO, (8) DA and a, b, c peaks corresponding to non-OPD derived GlcN condensation products.

**Fig 5:**
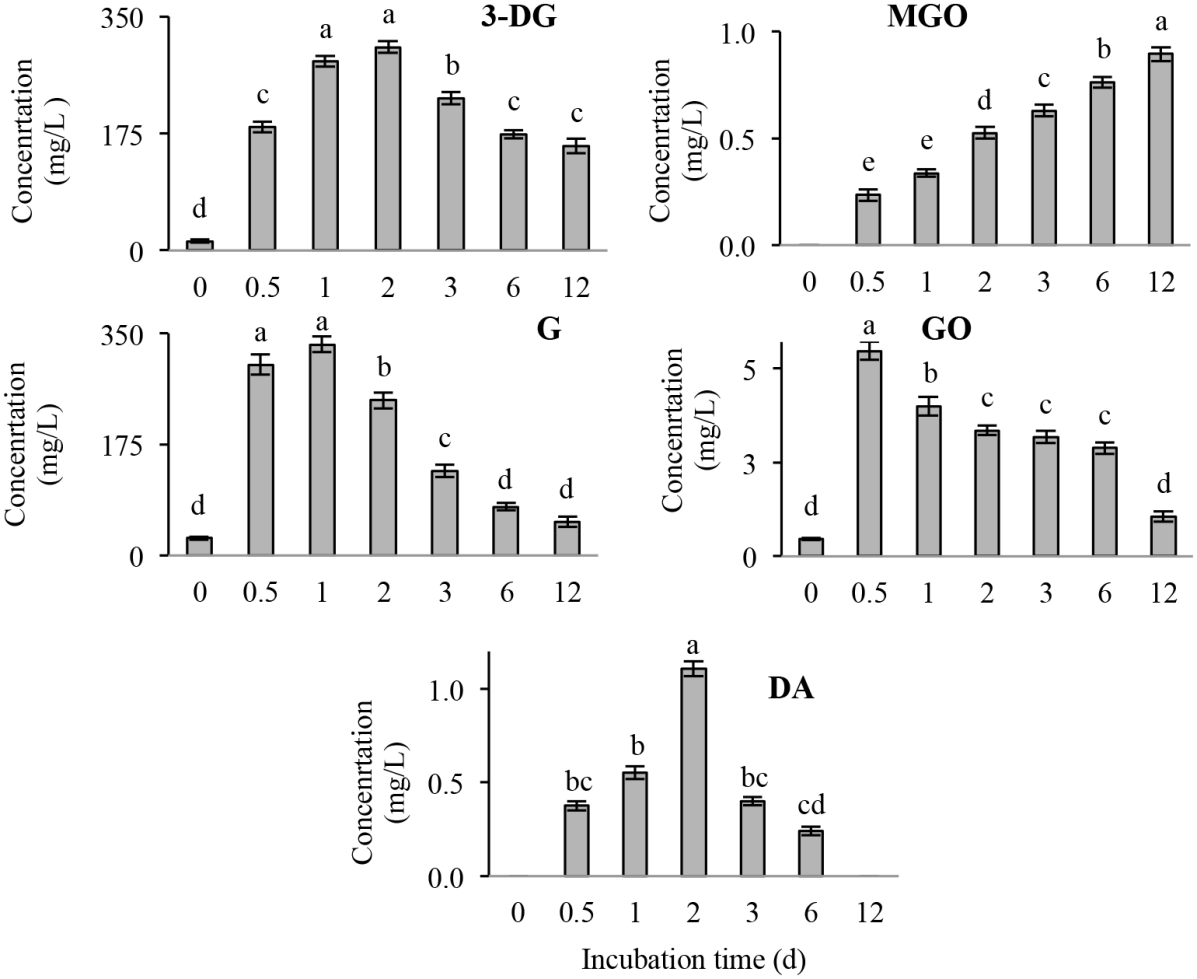
Concentration of the major α-dicarbonyl compound produced during incubation of Mb in the presence of GlcN from 0 to 12 days. The values are represented as mean ± standard deviation (calculated from three independent trials). G, glucosone; 3-DG, 3-deoxyglucosone; GO, glyoxal; MGO, methylglyoxal; DA, diacetyl. Different letters within each α-dicarbonyl compound indicate statistical significant difference (*p* < 0.05).

In a previous study [15] a significant formation of reactive α-DC from GlcN incubated at 37°C was reported. α-DC are up to 20,000-fold more reactive than Glc in inducing the Maillard reaction [40], which can explain the characteristic mass profile reported in Figs 1 and 2 for Mb-GlcN treatment. To investigate the amount of α-DC formed in Mb-GlcNAc/Glc/GlcN conjugates, the latter were trapped by reacting with *o*-OPD and the resulting quinoxaline derivatives were analyzed. However, there was almost no detectable α-DCs in Mb-GlcNAc conjugates up to 12 days of incubation, whereas α-DC were observed in Mb-Glc samples at 6 and 12 days of reaction (S6 Fig), but at a lower level as compared to Mb-GlcN. Hence, attention was devoted mainly on identifying and quantifying the α-DC derived from the Mb-GlcN reaction mixture. Fig 4B illustrates a UHPLC-UV representative chromatogram of Mb-GlcN conjugates incubated for 1 day. The identity of the peaks, determined by elution time, molecular mass and mass fragmentation pattern as compared to the standards (Fig 4A) and /or literature [18,19,41] is reported in Table 1. As expected, the quinoxaline derivative of G eluted first as identified at m/z 251.1038. It was followed by the peak (at m/z 235.0978) which could correspond to 1- or 4-deoxyglucosone, or 3-deoxygalactosone. The quinoxaline derivatives of 3-DG were identified at m/z 235.0991. GO derivatives eluted following HPA, 3,4-DGE, MGO and DA quinoxalines. After identifying the presence of major α-DC, five of them were quantified and the concentrations expressed in mg/L and are reported in Fig 5. G, 3-DG and GO were already present in control samples, suggesting a partial degradation of GlcN while it was stored in powder form. Previously the presence of G, 3-DG and GO was also found in untreated GlcN [15]. Overall, G and 3-DG were the most abundant α-DC in Mb-GlcN conjugates, followed by GO, MGO and DA. At the beginning of the incubation, G was the most abundant α-DC reaching the highest concentration at 0.5 and 1 day of the reaction (~332 mg/L on average). With prolonged reaction times its concentration dropped significantly (*p* < 0.05). 3-DG was the most abundant at 1 and 2 days of reaction, then decreased almost 2-fold by the end of the incubation time tested.

**Table 1:** Retention time, MS and MS/MS data of the α-dicarbonyl compounds detected Mb-GlcN conjugates.

Peak	Retention time (min)	Dicarbonyl compound (quinoxaine derivative)	Production (M+H)^+^	MS/MS fragments
1	21.3	Glucosone	251.1038	251.0930 (2), 233.0923 (100), 215.0817 (30), 173.0710 (32), 161.0710 (15), 145.0553 (2)
2	28.5	Unidentified	235.0978	235.0977 (5), 217.1944 (20), 199.0865 (10), 187.0864 (21), 179.1057 (45), 175.1474 (100), 157.0774 (8), 145.0759 (10)
3	32.7	3-Deoxyglucosone	235.0991	235.0992 (10),217.0974 (100),199.0868 (52), 187.0867 (3), 171.0917 (5), 157.0760 (12), 145.0760 (7), 130.0437 (4)
4	66.5	Glyoxal	131.0614	131.0601 (100), 104.0492 (38),102.4872 (12), 77.0163 (10)
5	70.7	Hydroxypyruvaldehyde	161.0722	161.0711 (7), 143.0602 (17), 139.1301 (10), 133.0760 (100), 119.0602 (87), 102.5203 (45), 71.1806 (12)
6	76.7	3,4-Dideoxyglucosone-3-ene	217.1712	217.1702 (100), 199.0869 (7), 181.1072 (7) 157.1234 (17), 145.0760 (7), 130.0729 (5)
7	79.8	Methylglyoxal	145.0773	145.0760 (100), 118 (12), 102.5180 (20), 77.2856 (5)
8	87.3	Diacetyl	159.0933	159.0998 (15), 145.0932 (12), 131.0603 (100), 102.4846 (47)

At shorter reaction times (0.5 and 1 d) the amount of G was 1.3 times higher than 3-DG, however with prolonged times to 6 and 12 days 3-DG became more prevalent by 2.2-fold. This suggests that at the beginning of the reaction the oxidative pathway is the dominant, and by increasing the reaction time the enolization pathway becomes favourable. A similar trend was reported for GlcN incubation alone with similar conditions [15], where the values for 3-DG were significantly (*p* < 0.05) lower for all studied times, except for 12 days, where the quantity of 3-DG reached 209 mg/L. When comparing G amount in GlcN incubated alone or Mb-GlcN conjugate at all incubation times, the presence of protein increased the amount of G produced.

The opposite relationship was found between GO and MGO generation over time in the Mb-GlcN system. While the concentration of GO significantly (*p* < 0.05) decreased from 5.5 mg/L at 1 day to 1.1 mg/L after 12 d, the amount of MGO significantly (*p* < 0.05) increased from 0.2 to 0.9 mg/L. MGO derives from fragmentation of 3-DG by a retroaldol condensation reaction [42], whereas GO is produced at the very early stages of the Maillard reaction or as a result of C-2/C-3 scission of the G [43].

For instance, at 0.5 day MGO accounted for 0.1% of 3-DG, whereas with incubation up to 12 days the percentage increased to 0.6. In comparison, MGO and GO derived from GlcN incubation was reported [15] to follow a similar tendency of increasing and decreasing over time, respectively, however in quantities much lower than in Mb-GlcN conjugate.

DA reached the highest concentration at 2 days of incubation (1.1 mg/L), followed by a significant (*p* < 0.05) decrease by 6 days and was not detected at 12 days of incubation. A similar trend of the highest amount at 2 days was observed for DA produced from GlcN incubation, but with a concentration around 3.2-fold less as in Mb-GlcN conjugate.

**Fig 6:**
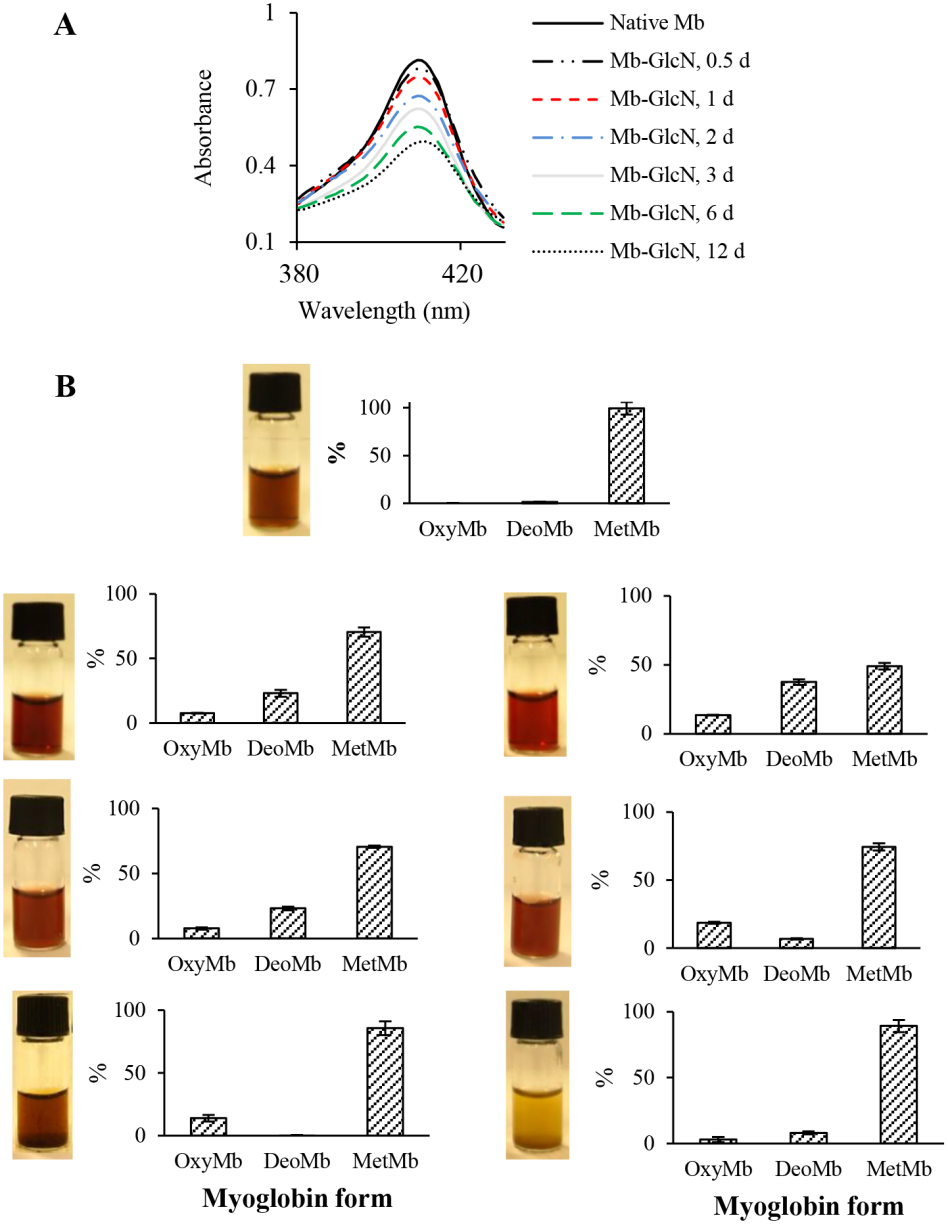
(A) Decrease in Soret band absorbance; (B) images and the percentages of metmyoglobin (MetMb), deoxymyoglobin (DeoMb) and oxymyoglobin (OxyMb) in native Mb and Mb-GlcN conjugates over glycation period from 0.5 to 12 days.

In summary, the amount of α-DC produced in Mb-GlcN, particularly at the beginning of the reaction, was greater compared to the one observed in non-enzymatic degradation of GlcN alone [15]. The reason for this phenomenon is likely due to Mb supplying side chain amino groups for the Maillard reaction with GlcN; however it may also be due to the catalytic effect of heme iron on both GlcN and Amadori product oxidative degradation. In this regard, as shown in Fig 6A about the Soret band, heme iron was displaced from the hydrophobic core of Mb during the course of glycation. This would increase its ability to catalyze the oxidation degradation of GlcN and Amadori intermediates to α-DC. Further investigations are required to confirm this phenomenon.

### Mb-Fe^3+^-reducing activity of GlcN

Of interest is the effect of GlcN on the oxidative status of iron in Mb (Fig 6). After 1 day of incubation the proportion of Mb-Fe^2+^ (deoxy- + oxyMb forms) relative to Mb-Fe^3+^ (metMb) increased by up to 50%. There are several theories to explain this phenomenon. For instance, Stepuro et al. [44] observed reduction of ferriforms of cytochrome C and metMb by adding glycated amino acids or glycated albumin. The authors proposed it was due to the effects of a combination of O_2_^-^ and α-dicarbonyls in the reduction process. Gersten et al. [45] reported that the reaction of bovine cytochrome C and R5P rapidly generated superoxide (O_2_^-^) that subsequently reduced ferri- to ferro-cytochrome C. Also these authors noted that the addition of amines to the cytochrome C-R5P system greatly increased the generation of O_2_^-^ at 37°C. The reaction of R5P with metMb also resulted in the formation of oxyMb within 1–2 days of the reaction at 37°C and was ascribed to the rapid generation of O_2_^-^ from sugar which subsequently reduced Mb`s state. Glycation can also generate “reductones”, which refers to compounds with an enediol structure next to a carbonyl, such as G [46]. As a matter of interest, the production of G which was the highest one at 1 day (Fig 5), however, with time, this effect was lost and the deleterious effect of α-DC and ROS (i.e. H_2_0_2_) on Mb structure became dominant. Finally, in the last stage of the Maillard reaction free and bound AGEs to protein are formed. Some AGEs, like for instance argpyrimidine, have been recognized to act as reducing agent inducing formation of oxyMb in fructated Mb [47]. In addition, dihydroxyfructosazines deriving from GlcN cyclocondensation were also reported to carry a reducing power [48].

### Conformational changes of myoglobin during glycation in the presence of glucosamine

#### Intrinsic fluorescence of glycated Mb

**Fig 7:**
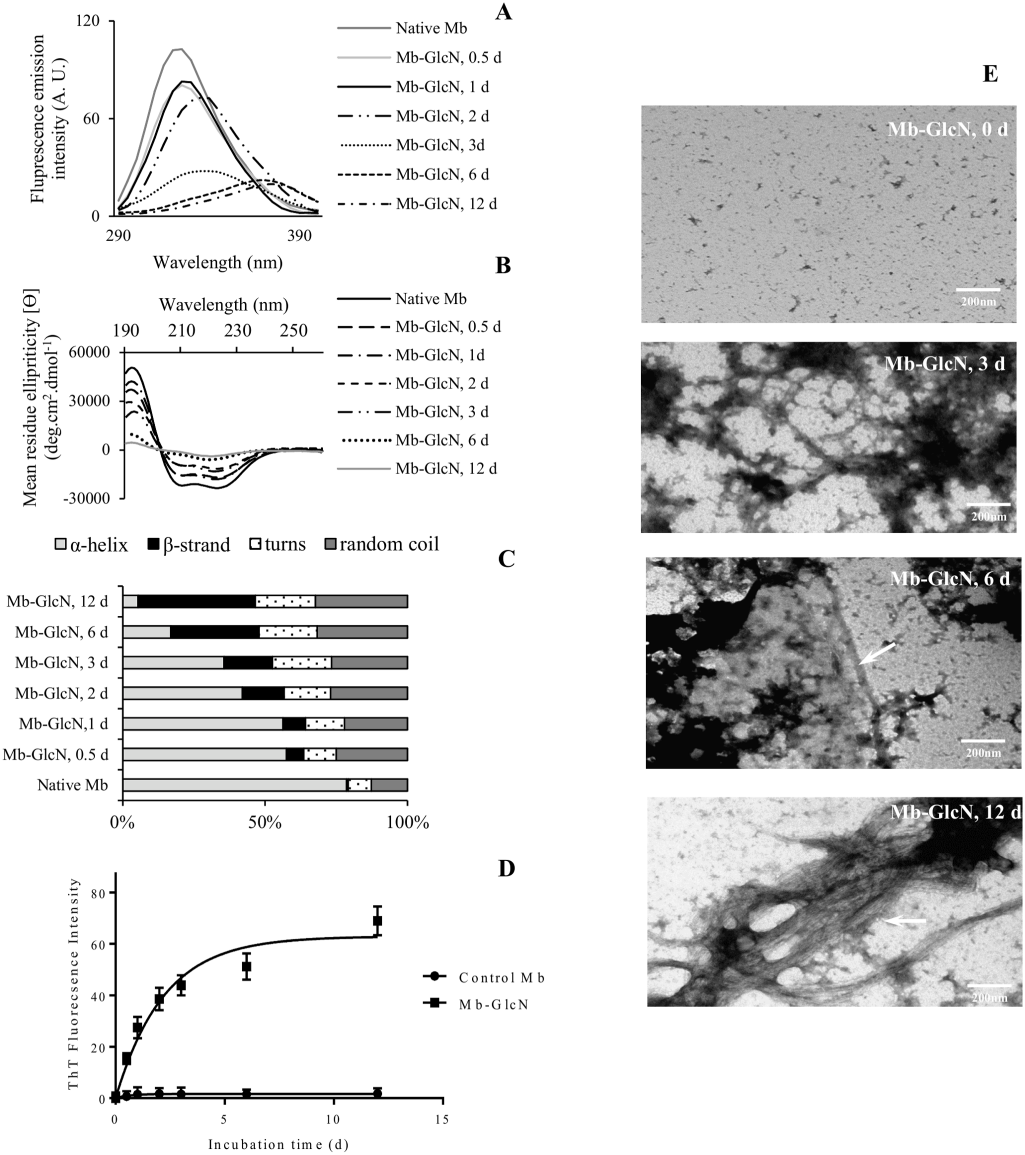
(A) Fluorescence emission spectra; (B) far-UV spectra analyses; (C) secondary structure composition; (D) maximum fluorescence intensity of Thioflavin T (λ = 482 nm). The data points all have SD bars, but some are illegible and lie within the symbols; (E) transmission electron micrographs of native Mb and Mb incubated in the presence of GlcN at specific time points.

Fluorescence spectroscopy analyses were performed to examine the alterations in the molecular structure of glycated Mb. Mb-GlcNAc treatments did not show any significant impact on the protein structural arrangement. Even though glycation with Glc was found to occur, particularly at longer reaction times, the effect on conformation was significantly less as compared to GlcN. The following discussion focuses on the structural modification of Mb induced by GlcN. When excited at 280 nm, the protein tertiary structure is reflected, since this frequency of electromagnetic radiation excites Trp and Tyr. In the region of 290 to 400 nm, native Mb has one emission peak with a maximum intensity at 325 (Fig 7A). Glycation markedly decreased emission intensity in relation to the native protein, after 0.5 days of reaction. This suggests a strong influence of GlcN attachment on the local environment around Trp and Tyr residues. Even though changes in intrinsic fluorescence are symptomatic to the local changes in the Trp and Trp microenvironment, they are usually associated with larger structural rearrangements of the peptide chains, including protein polymerization. Mb contains two highly conserved tryptophan residues located at positions 7 and 14 on the α-helix [49]. According to Tang et al. [50] attachment of sugar moieties to protein causes Trp hydrophobic chromophores to become more buried within the molecules, resulting in a lower extent of interaction with quenching agents. Indeed, in folded Mb the Trp residues are partially quenched by the heme group [51]. Consequently, a decrease in Trp fluorescence in glycated Mb could correspond to a more closed structure where the Trp is closer to the heme group, increasing its quenching effect. On the other hand, Swamy and Surolia [52] proposed that decrease in fluorescence results from the exposure of tryptophanyl residues to the solvent molecules that collide with fluorophores and consume fluorescence energy. The decrease could also be induced by oxidation, since the indole ring of Trp and side chain of Tyr are susceptible to oxidative damage by ROS [53].

Upon incubation, the decrease in emission was accompanied by a progressive bathochromic shift (~15 nm) in the wavelength of maximum emission (λ_max_). This shift usually corresponds to an exposure of intrinsic tryptophan residues of glycated protein to a more polar medium in the presence of carbohydrates [54]. Fluorescence emission of Trp7 is significantly quenched by Lys79 (EF2 helix), therefore a red shift in emission could be also due to the movement of this Lys away from Trp7 when the α-helix unfolds [55] as a result of glycation. At the same time glycation-induced conformational changes could increase the separation between two of Mb’s Tyr residues (Tyr103 and 146) and energy acceptors, especially for Tyr 103, which is located in a close proximity to the heme. The same results of decreasing in fluorescence intensity along with a red shift were reported in the study of Tang et al. [50], who performed glycation of phaseolin with Glc at 60°C. Another study by Jin et al. [56] also reported decrease in fluorescence concomitantly with red shift while investigating the deactivation of chloroperoxidase by different monosaccharide, attributing this to the uncoiling of α-helical structures in the presence of sugar and protein precipitation. In the current study, strong protein precipitation was observed when glycating with GlcN, observed at 6 days of reaction (Fig 7E). This remarkable change in fluorescence emission suggests that glycation with GlcN causes substantial alterations in Mb`s tertiary structure along with displacement of heme from the hydrophobic pocket within Mb’s interior (Fig 6A).

#### Secondary structure fingerprint by circular dichroism spectropolarimetry

After confirming changes in tertiary conformation of Mb, it was attempted to detect whether the secondary structure was altered during glycation. The far-UV (190–260 nm) CD spectrum is an extremely sensitive probe for protein secondary structure, reflecting its structural features due to absorption of the peptide bond [57]. The conjugation of sugar induced the changes in the secondary structure of Mb, as shown by the difference in CD spectra of native and glycated protein in far-UV CD region (Fig 7B). The CD spectra of native Mb (control) displayed CD bands in a wavelength shorter than 240 nm, which is characterized by two negative bands (210 and 222 nm) and single positive (192 nm). The secondary structure of native Mb was 78.6% α-helix, 0.6% β-strand, 8.1% turns and 12.7% random coil (Fig 7C). Horse myoglobin consists of eight helical regions (named A to H), separated by non-helical segments (AB, BC, CD…GH) [58]. Compared to native Mb, Mb-GlcN conjugates showed a gradual decrease in negative ellipticity in the region 210–225 nm, associated with a decrease in α-helix content (Fig 7C). This decrease in α-helix content of glycated Mb was compensated by an increase in β-sheets, random coiling and turns, suggesting the disruption of secondary structure by glycation. As shown (Fig 7B and 7C) glycation over time greatly impacted the secondary conformation of protein, evident already at 0.5 days. This corresponds to information obtained from ESI-MS, where the changes in Mb-GlcN conjugates were identifiable already at 0.5 days of glycation. Such major modifications of secondary structure composition over time result from the steric hindrance attained from the attachment of GlcN and/or its derivatives. They could be involved in protein unfolding and subsequent aggregation by the formation of intermolecular β-sheets. In 12 d, only 5.4% of Mb`s α-helical structure retained, while 41% of its chains attained a β-sheet conformation. This increased tendency to form highly insoluble fibrillar aggregates during glycation of an original α-helical structure into a predominantly β-sheet conformation was reported previously for different proteins, including apoMb [59], albumin [60,61] and islet [62]. Uzzan et al. [63] in an attempt to produce a GlcN-enriched milk beverage reported that GlcN instantaneously destabilized the milk protein system at 100°C or higher, causing a rapid and drastic precipitation. In a search for evidence of a β-sheet conformation, implying the formation of amyloid-like aggregates, the glycated Mb preparations were analyzed by transmission electron microscopy and thioflavin T staining.

#### Aggregation propensity of Mb-GlcN conjugates: ThT fluorescence and TEM

In order to understand if aggregates formed in the presence of GlcN were amyloidogenic, a benzothiazole fluorescent dye (ThT) was added to the samples. ThT, as an extrinsic fluorescent probe molecule, once binding to amyloid fibrils, gives a characteristic fluorescent emission at ~480 nm with intensity proportional to the amount of amyloid fibrils present [64]. As shown in Fig 7D, the fluorescence of ThT at 482 nm slightly increased at 0.5 days incubation relative to the Mb control, whereas a marked increase was observed after 2 days. At a value of 62.9 the fluorescence intensity reached a plateau and remained almost unchanged up to 12 days of incubation. This suggests an onset of fibril formation. At the same time, Mb incubated with GlcNAc and Glc (S7 Fig) showed no significant changes in ThT fluorescence under the experimental conditions applied. The control Mb showed no changes in fluorescence intensity at 482 nm when exposed to the dye, and therefore Mb did not bind the dye. (Fig 7D). The GlcN-induced modification accelerated Mb aggregation and promoted the formation of amyloid fibrils. In addition, recent studies suggest that some protein molten globules are positive in ThT fluorescence. The increase at the beginning could correspond to a molten globular structure of Mb which over time transforms into fibril-like structures.

Using TEM the morphologies of Mb-GlcN aggregates were examined over time (Fig 7D). Amorphous aggregates, possibly pre-fibrillar species, were observed at 3 days of incubation. Prolonged incubation up to 6 and 12 days resulted in observations of mature bundles of fibrous structures (indicated by arrows). Mb glycation with GlcN strongly affected aggregation behaviour of protein, producing amyloid fibrils within 6–12 days. This agrees with previous studies where glycation with D-ribose promoted rapid amyloid-like aggregation of bovine serum albumin [65], Tau [66] and α-Synuclein [67]. Sirangelo et al. [49] demonstrated that substitutions of both of apoMb’s tryptophan residues endowed it with a high propensity to rapidly form fibrils under physiological conditions. This is corroborated with the fluorescence results (Fig 7A), where a significant decrease was associated with changes in the environment of Trp and Tyr amino acid residues.

A specific sequence of events occur to induce the formation of amyloid in Mb, which involves modifications of amino groups at the exposed side of protein, that then stimulates its refolding from a globular state to fibrillar. Firstly, this could occur by the covalent binding of GlcN and derivatives (i.e. α-DC) to amino acids in a way that alters their environment causing partial unfolding. Another mechanism would be through extreme cross-linking due to the production of α-DC. For instance, 3-DG rapidly reacts with protein amino groups to form AGEs, including imidazolone and pyrraline [68]. MGO and GO also react with proteins rapidly and directly producing hydroimidazolones and variety of lysine-derived adducts [69]. Lastly, numerous inter- and intramolecular AGE-bridged cross-linkages can exert mechanical stress on the polypeptide chain that facilitates its unfolding [60]. For instance, AGE-induced cross-linking of collagen during incubation with glucose at 37°C was shown by Sajithlal et al. [70]. This variety of cross-links may promote diversity of new bonds between newly-exposed amino acids stimulating the formation of β-sheet structure, a prerequisite for amyloid fibril formation [71]. The multimerization or condensation of glycated proteins into plaque has been reported for extracellular matrix proteins (i.e. fibrinogen and collagen) [72]. In this way, α-DC and AGEs act as denaturants bringing together sequences that have a propensity to fold into β-sheet structure.

## Conclusions

This study provides further evidence that GlcN is a highly reactive monosaccharide, leading to heme reduction, displacement and significant modifications of protein conformation, involving fragmentation and formation of amyloidal-type fibrils. Production of α-DC and AGEs are suggested to play a primary role in protein aggregation rapidly observed after 3 days of reaction. At the same time, GlcN`s acetylated counterpart, GlcNAc, did not induce significant changes to protein conformation within the timeline studied, confirming the involvement of -NH_2_ group with the increased rate of modification.

## Supporting Information

S1 Fig Blocked amino groups upon glycation of Mb with GlcNAc, Glc and GlcN through the Maillard reaction for various incubation periods (0.5, 1, 2, 3, 6 and 12 d). (TIFF)

S2 Fig Total (A) and free (B) AGEs in Mb-GlcNAc, Mb-Glc and Mb- GlcN mixtures incubated for various periods (0.5, 1, 2, 3, 6 and 12 d) at 37°C. (TIFF)

S3 Fig Multiple-charged ions spectrum of Mb-GlcNAc control and incubated mixtures acquired on an Agilent 6220 ESI-TOF MS. A) Full spectrum at 0 days. B) Zoomed in spectrum at Z = 21–18. C) Full spectrum at 12 days. D) Zoomed in spectrum at Z = 21–18.
(TIFF)

S4 Fig Multiple-charged ions spectrum of Mb-Glc control and incubated mixtures acquired on an Agilent 6220 ESI-TOF MS. A) Full spectrum at 0 days. B) Zoomed in spectrum at Z = 21–18. C) Full spectrum at 12 days. D) Zoomed in spectrum at Z = 21–18.
(TIFF)

S5 Fig Multiple-charged ions spectrum of Mb-GlcN control and incubated mixtures acquired on an Agilent 6220 ESI-TOF MS. A) Full spectrum at 0 days. B) Zoomed in spectrum at Z = 21–18. C) Full spectrum at 6 days. D) Zoomed in spectrum at Z = 21–18.
(TIFF)

S6 Fig UHPLC chromatograms of (A) Mb-GlcNAc incubated for 12 days; (B) Mb-Glc and (C) Mb-Glc incubated for 6 and 12 days, respectively, derivatized with *o*-phenylenediamine and recorded at 314 nm. (TIF)

S7 Fig The mean of triplicate Thioflavin T fluorescence measurements (λ = 482 nm) in Mb control, Mb-Glc and Mb-GlcNAc treatments .
(TIFF)
