# Supplementary figures and images for: Rapid Myoglobin Aggregation through Glucosamine-Induced α-Dicarbonyl Formation
Source: PLoS One. 2015 Sep 25;10(9):e0139022. doi: 10.1371/journal.pone.0139022 (PMC4583429; doi:10.1371/journal.pone.0139022)

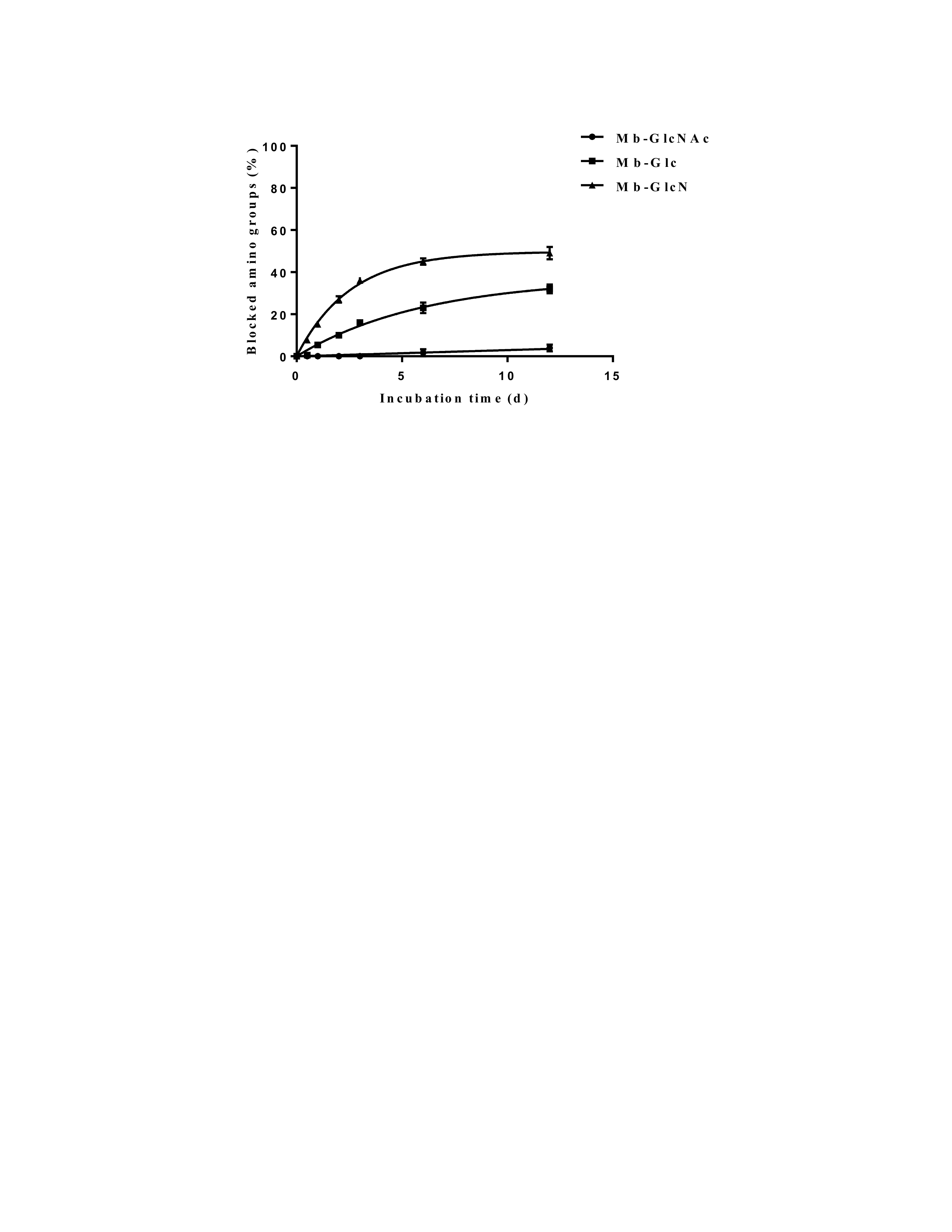

Supplement: S1 Fig — (TIFF) [file pone.0139022.s001.tiff]

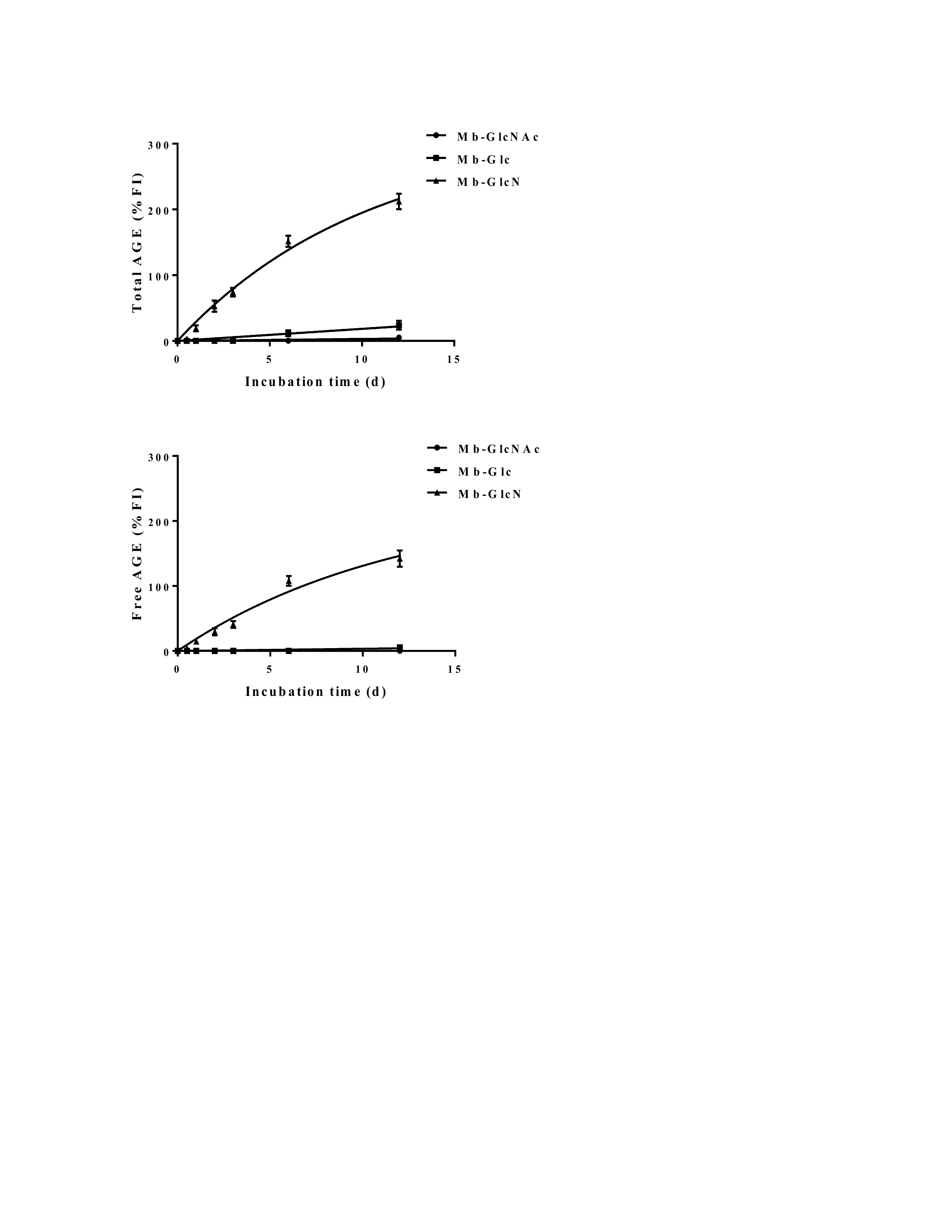

Supplement: S2 Fig — (TIFF) [file pone.0139022.s002.tiff]

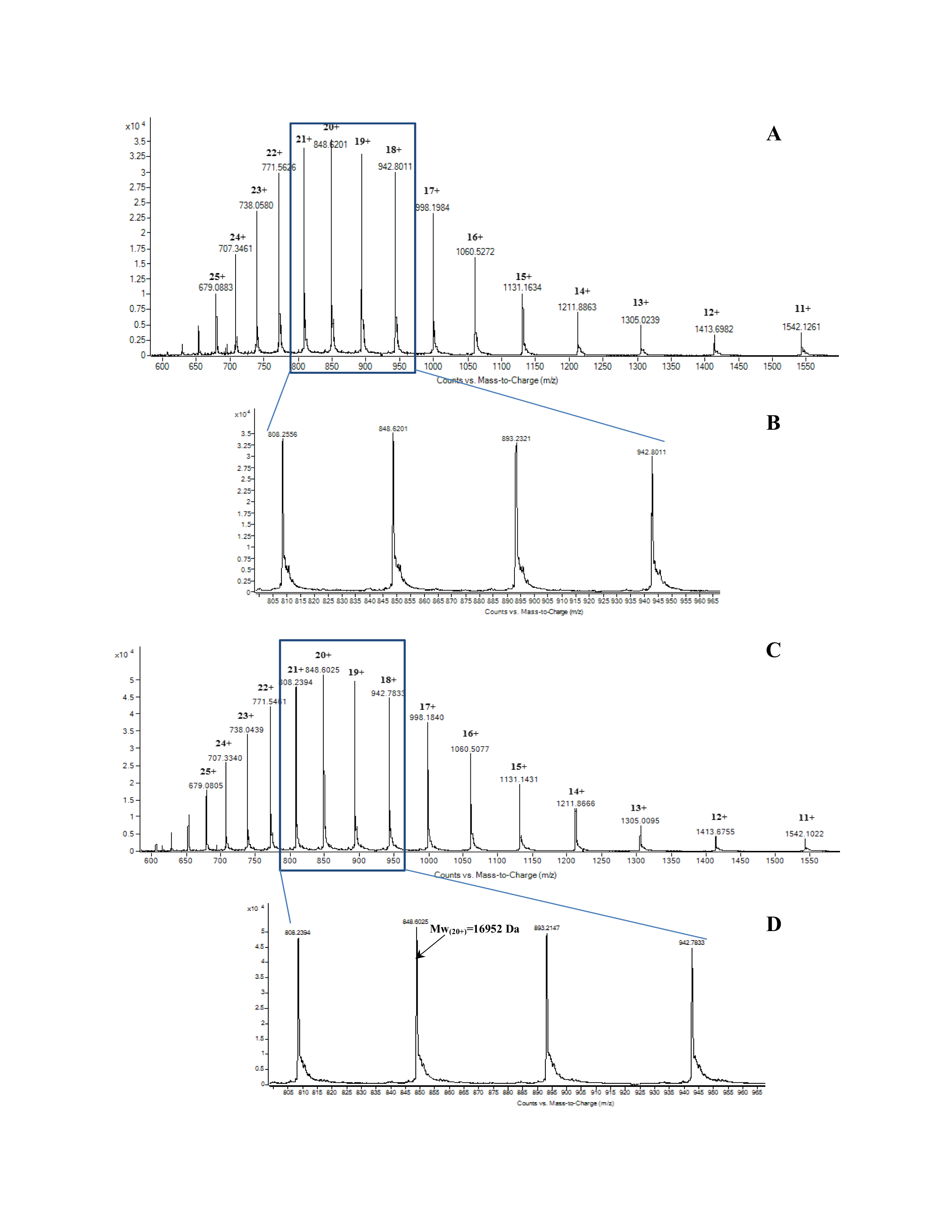

Supplement: S3 Fig — A) Full spectrum at 0 days. B) Zoomed in spectrum at Z = 21–18. C) Full spectrum at 12 days. D) Zoomed in spectrum at Z = 21–18. (TIFF) [file pone.0139022.s003.tiff]

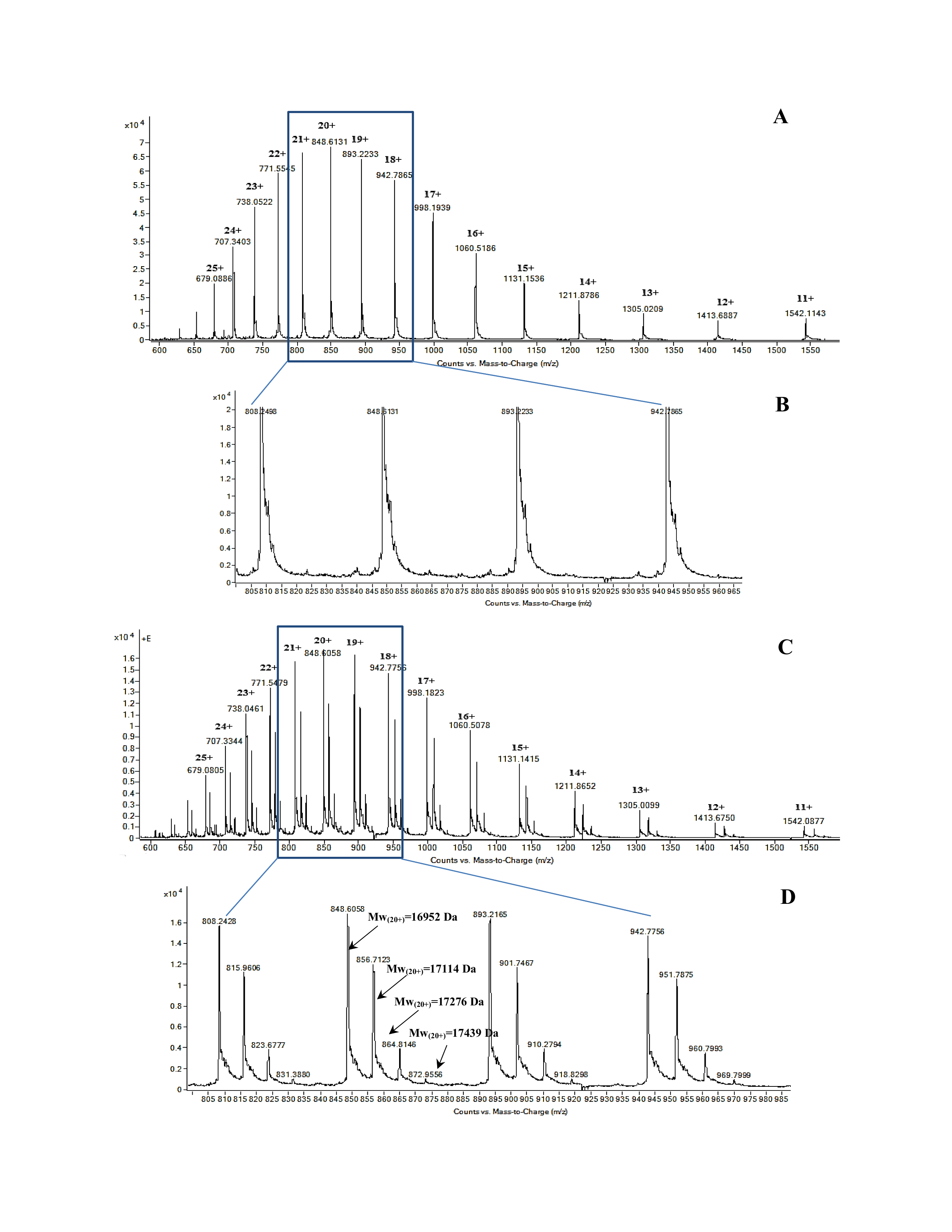

Supplement: S4 Fig — A) Full spectrum at 0 days. B) Zoomed in spectrum at Z = 21–18. C) Full spectrum at 12 days. D) Zoomed in spectrum at Z = 21–18. (TIFF) [file pone.0139022.s004.tiff]

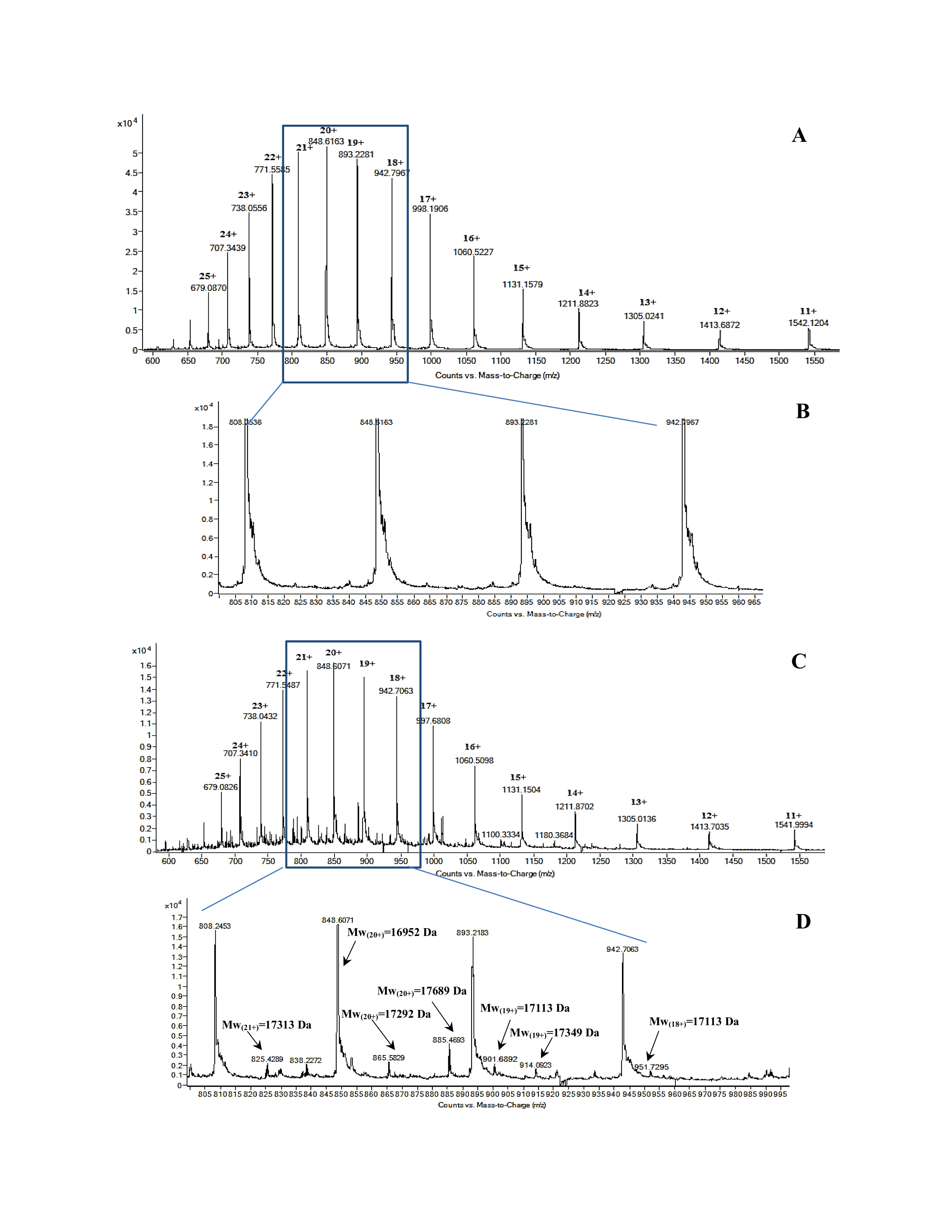

Supplement: S5 Fig — A) Full spectrum at 0 days. B) Zoomed in spectrum at Z = 21–18. C) Full spectrum at 6 days. D) Zoomed in spectrum at Z = 21–18. (TIFF) [file pone.0139022.s005.tiff]

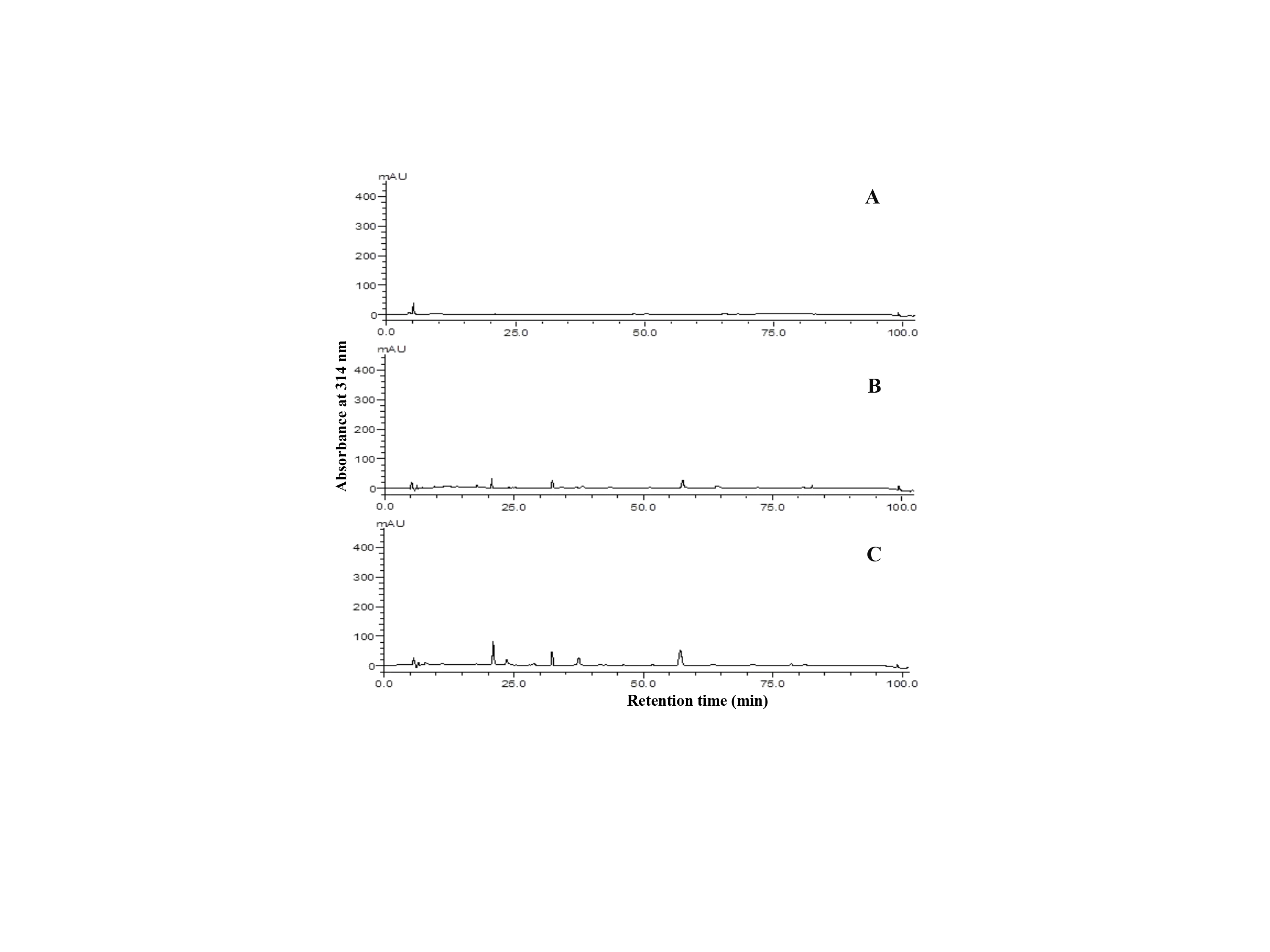

Supplement: S6 Fig — (TIF) [file pone.0139022.s006.tif]

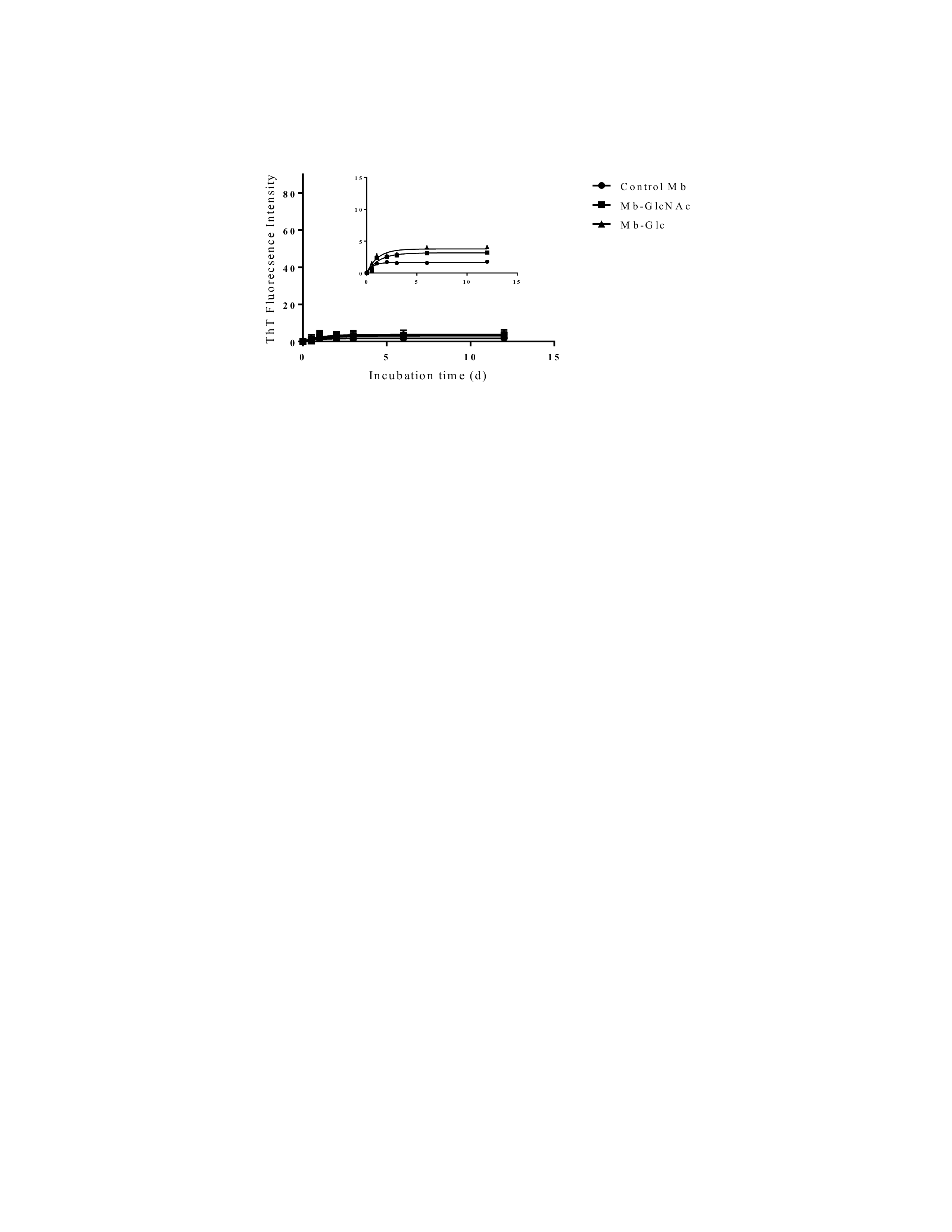

Supplement: S7 Fig — . (TIFF) [file pone.0139022.s007.tiff]
